# Decision-Making in Refractory Overactive Bladder: Evaluation of a Novel Patient Decision Aid and its Impact on Decisional Conflict

**DOI:** 10.1007/s00192-026-06688-3

**Published:** 2026-05-19

**Authors:** Rayan Mohamed-Ahmed, Angie Rantell, Annika Taithongchai, George Araklitis, Dudley Robinson

**Affiliations:** Department of Urogynaecology, King’s College Hospital, Denmark Hill, London, SE5 9RS UK

**Keywords:** Overactive bladder, Refractory overactive bladder, Patient decision aid, Shared decision-making

## Abstract

**Background:**

Refractory overactive bladder (OAB) lacks formal definition and is poorly understood. Its management requires patients to choose an invasive treatment, including intravesical botulinum toxin A (BOTOX®), sacral neuromodulation (SNM) and posterior tibial nerve stimulation (PTNS), which have varying rates of efficacy and side effects. These decisions can be complex for patients to make, especially within the time constraints of outpatient clinic appointments. A new patient decision aid (PDA) has been developed to support patients during this shared decision-making process.

**Aim:**

This study assesses decisional conflict scores and patient satisfaction in those who use a novel PDA for refractory OAB.

**Methods:**

Consecutive new patients with refractory OAB were provided with the PDA prior to decision-making. Patients then had a virtual follow-up appointment to assess their decisional conflict, using a validated scale. The total decisional conflict score was calculated. Treatment choice and free-text feedback were also analysed.

**Results:**

Twenty-five patients were included. Following use of the PDA, a range of treatment choices were made, including SNM (*n* = 6), PTNS (*n* = 10), BOTOX® (*n* = 6) and continuation with medical therapy (*n* = 3). Overall, decisional conflict was low, with 100% of patients having a score of less than 25. Subscores suggested improved patient understanding, with the PDA increasing clarification.

**Conclusion:**

Introduction of this novel PDA, in those requiring treatment for refractory OAB, was associated with low decisional conflict and positive patient feedback. This supports its use in shared decision-making, empowering patients to make an informed and individualised choice regarding their treatment.

## Introduction

Overactive bladder (OAB) is a common and distressing condition characterised by urinary urgency, usually accompanied by frequency and nocturia, with or without urgency urinary incontinence, in the absence of urinary tract infection or other obvious pathology [1]. Although first-line conservative measures and pharmacological treatments are effective for some patients, a significant proportion experience persistent symptoms despite optimal conservative and medical management [2, 3]. These patients are often described as having refractory OAB, although no universally accepted definition exists.

Management of refractory OAB typically requires patients to consider invasive or device-based therapies, including intravesical botulinum toxin A injections, sacral neuromodulation (SNM) or posterior tibial nerve stimulation (PTNS), as recommended by the National Institute for Health Care and Excellence in the UK (NICE) [2]. Each option has distinct mechanisms of action, efficacy profiles, risks, reversibility and treatment burdens. For example, botulinum toxin A is effective but associated with risks of urinary retention and urinary tract infection [4, 5]; SNM offers durable symptom improvement but involves implanted hardware and potential revision surgery [6, 7]; PTNS is minimally invasive but requires repeated clinic visits and ongoing maintenance treatments [8]. Implantable tibial neuromodulation devices, including the eCoin or BlueWind devices, show promising results, although they have not yet been approved for clinical use in the UK [9, 10].

Choosing between these options can therefore be a difficult decision for an individual to make and there is no ‘one size fits all’ approach.

Shared decision-making (SDM) is increasingly recognised as a cornerstone of high-quality, patient-centred care, particularly where multiple reasonable treatment options exist and no single option is clearly superior [11]. It requires collaboration between a clinician and a patient, to allow patient understanding of a disease and the possible treatment options and through such discussion, allows a patient to come to a conclusion regarding the treatment choice that best suits them.

An integral aspect of SDM, is the use of patient tools to support decision-making [11]. Patient decision aids (PDAs) are structured tools designed to support this process by providing balanced, evidence-based information about available options and helping patients clarify their values and preferences [12]. PDAs have been shown across multiple specialities to improve patient knowledge, reduce decisional conflict and increase alignment between patient values and chosen treatments [13–15]. Decisional conflict is defined as ‘personal uncertainty about which course of action to take when choice among competing options involves risk, regret, or challenge to personal life values’ [16]. This can be influenced by lack of adequate information regarding the decision as well as inadequate support.

In urogynaecology, PDAs have been more extensively studied in the context of stress urinary incontinence and pelvic organ prolapse surgery, where their use has been associated with reduced decisional conflict and improved patient satisfaction [17–19]. However, evidence supporting the use of PDAs in refractory OAB is limited. A computerised PDA for treatment of OAB was assessed within a population of 489 patients with urgency and urgency incontinence, or urge predominant mixed urinary incontinence, which demonstrated the benefit of use of a PDA in this clinical context [12]. It also demonstrated that patients may prefer to access treatment in individualised manners, rather than following generic treatment protocols. Given the increasing complexity of OAB treatment pathways, the long-term nature of treatment and the time constraints of outpatient clinics, there is a clear need to evaluate tools that may enhance patient understanding and engagement in decision-making.

A novel PDA for refractory OAB has recently been published by a group of international clinicians and patient societies, to support patients considering invasive second line treatment and assist healthcare professionals when counselling patients on all available treatment options [20]. The PDA is a 39 page document, including appendices and references, which discusses the background of OAB, the different treatment options for refractory OAB, how a patient may choose the right treatment for them and where to access further support. The PDA was developed with the aid of an unrestricted educational grant from Medtronic, with its creation managed independently by a third-party vendor and the clinician steering group to derive appropriate clinical content. The PDA has been supported by several societies, including the International Continence Society (ICS) and European Association of Urology (EAU). None of the authors were involved in the development of this PDA. This study aimed to evaluate the impact of this PDA on decisional conflict and patient satisfaction in women with refractory OAB, using a validated outcome measure.

## Methods

### Study Design and Setting

This prospective observational cohort study was conducted within a tertiary urogynaecology service in the UK.

### Participants

Consecutive women attending the urogynaecology service with refractory OAB were invited to participate. Refractory OAB was defined pragmatically as persistent OAB symptoms despite adequate trials of conservative measures, such as bladder re-training and pelvic floor muscle training, and at least one pharmacological treatment for 4 months. Eligible participants were considering further management options, including intravesical botulinum toxin A, SNM or PTNS.

Patients were excluded if they were unable to engage with the PDA due to cognitive impairment, language barriers or if specific treatment options were clinically contraindicated, thereby limiting genuine choice. Demographic data and relevant clinical information were collected from medical records.

### Intervention: Patient Decision Aid

Participants were provided with access to the PDA prior to making a treatment decision. The treatment options were also discussed in a clinic appointment when the PDA was provided. This was provided in a paper as well as electronic format with a QR code. The PDA presented information on refractory OAB and outlined the available treatment options which were Botox, SNM and PTNS, including mechanisms of action, benefits, risks, side effects, practical considerations and likely follow-up requirements. It then encourages patients to consider the impact of their OAB symptoms and what matters to them with regard to their treatment choice. Patients were encouraged to review the PDA independently and, where appropriate, with family members or friends.

A virtual follow-up consultation was arranged 6 to 8 weeks later, to discuss treatment options after PDA review. This consultation formed part of routine clinical care.

### Outcome Measure: Decisional Conflict

Decisional conflict was assessed using the validated Decisional Conflict Scale (DCS) [21]. The DCS is a 16-item questionnaire that measures personal perceptions of uncertainty in choosing options, modifiable factors contributing to uncertainty and perceived effectiveness of decision-making. It comprises five subscales:

1. Informed: Perception of being informed about options.
2. Values clarity: Personal values integrated into the choice.
3. Support: Perceived support from providers and family.
4. Uncertainty: Personal uncertainty regarding the choice.
5. Effective decision-making: Satisfaction and confidence in the decision.

Each item is scored on a 5-point Likert scale. A total score from 0–100 is then calculated by adding up the total scores from each question, dividing by 16 and then multiplying by 25. Scores below 25 are associated with low decisional conflict and readiness to implement decisions, whereas scores above 37.5 are associated with decisional delay or uncertainty [21].

Participants completed the DCS following their PDA review and follow-up consultation. Treatment choice was recorded and free-text comments were invited to capture qualitative feedback.

### Data Analysis

Descriptive statistics were used to summarise participant characteristics, treatment choices and DCS scores. Mean and range of total DCS scores were calculated. These were performed using Excel. Subscale scores were analysed to explore domains contributing to residual decisional conflict. Qualitative free-text responses were reviewed thematically to contextualise quantitative findings.

## Results

### Participant Characteristics

Twenty-eight women with refractory OAB were included in the study, with a median age of 55 (range 32–69). There was a mixture of ethnicities, including ‘Black British’ (*n* = 9), ‘White British’ (*n* = 8), ‘White other’ (*n* = 5), ‘Asian’ (*n* = 2) and ‘Mixed’ (*n* = 1). All participants were able to engage fully with the PDA. Three patients were excluded following review of their treatment choice, due to relative contra-indications such as raised BMI and cognitive impairment. An alternative treatment choice was therefore recommended. The responses of 25 women were therefore analysed.

### Treatment Choices

Following use of the PDA, participants selected a range of management options reflective of individual preferences and values. PTNS was the most commonly chosen option (*n* = 10), followed by SNM (*n* = 6) and intravesical botulinum toxin A (*n* = 6). Three participants opted to continue with conservative or medical therapy, indicating that the PDA also supported informed decisions to defer invasive treatment.

### Decisional Conflict Scores

All participants (100%) reported total DCS scores below 25, indicating uniformly low decisional conflict. The mean total DCS score was 13.7, with scores ranging from 0 to 25. Subscale analysis demonstrated particularly low scores in the informed and values clarity domains, suggesting that the PDA effectively supported patient understanding and alignment of choices with personal priorities.

Uncertainty and effective decision-making subscales also demonstrated low scores, indicating high confidence in decisions made following PDA use.

### Qualitative Feedback

Free-text feedback was provided by the majority of participants and was overwhelmingly positive. Patients described the PDA as ‘more detailed than clinic discussions’ and ‘extremely helpful when reviewed with family’. Several participants highlighted that having access to structured information outside the clinic environment reduced pressure and allowed time for reflection.

Free-text responses provided insight into why the PDA was effective:

- **Detail and Accessibility:** Patients described the tool as ‘more detailed than clinic discussions’ and ‘easy to read’.
- **Social Support:** One patient noted the PDA was ‘extremely helpful when reviewed with family’ highlighting the importance of involving the patient’s support network in surgical decisions. Patients who described English as their second language also reported that ‘my family translated it for me so that I could talk it through with them’.
- **Visual Aids:** Responses indicated that ‘diagrams are good’ and the ‘setup is easy to understand’.
- **Clarity:** Patients reported that the PDA ‘clarified information’, was ‘easy to understand’ and ‘straightforward from a layman’s perspective’.
- **Information:** patients felt that the PDA was ‘very helpful’, ‘very informative’ and ‘interesting’.

## Discussion

### Main Findings

This study demonstrates that use of a novel PDA for refractory OAB is associated with uniformly low decisional conflict, with all participants reporting DCS scores below the threshold associated with decisional uncertainty or delay. Importantly, low decisional conflict was observed across all treatment choices, including invasive therapies and decisions to defer further intervention, suggesting that the PDA supported high-quality, decision-making rather than promoting procedural uptake.

### Interpretation in the Context of Existing Evidence

Decision-making in refractory OAB is particularly challenging because available second-line treatments differ substantially in invasiveness, reversibility, burden of follow-up and side-effect profiles, with no clear hierarchy of superiority. Previous studies evaluating PDAs in urogynaecology have focused largely on stress urinary incontinence and pelvic organ prolapse surgery, demonstrating reductions in decisional conflict and improved patient satisfaction (17, 18). Evidence for PDAs in OAB, however, has been limited.

The findings of this study extend existing evidence by demonstrating that PDAs are similarly effective in the context of refractory OAB, where decision-making can be complex. The mean DCS score observed in this cohort compares favourably with published PDA studies in pelvic floor surgery and other medical specialties, reinforcing the generalisability of PDAs as tools for supporting complex treatment decisions.

The subscale analysis suggested particularly low scores in the domains of ‘being informed’ and ‘values clarity’. This is clinically significant, as inadequate understanding and poor alignment with personal values are key drivers of decisional regret and dissatisfaction following invasive interventions. This may in turn lead to blaming healthcare professionals (22). By supporting patients to consider the individualised risks versus benefits, as well as potential impact on lifestyle, the PDA appears to facilitate decisions that patients feel confident implementing.

### Clinical and Service-Level Implications

Outpatient consultations for refractory OAB are frequently constrained by time, yet require detailed discussion of multiple complex treatment options. PDAs offer a pragmatic solution by allowing patients to engage with balanced information outside the clinic environment.

Previous evidence within the literature shows high discontinuation rates of invasive treatments, with long-term cohort studies showing only 30% of patients continue treatment with intravesical Botox and 20% of patients continuing with PTNS ‘top ups’ for long-term management of their refractory OAB (4, 23). Use of this PDA may allow patients to choose the most appropriate treatment for them in the first instance, which continues patient engagement with their treatment, rather than disengagement due to failed treatments. This may improve patient satisfaction and efficiency with the service.

### Strengths and Limitations

A major strength of this study is the use of a validated, internationally recognised scale (DCS) to quantify patient experience.

Limitations include the small sample size (*n* = 25) and the lack of a control group. Furthermore, as the PDA was only available in English, the results may not be generalisable to non-English speaking populations, where language barriers are known to impact surgical outcomes and comprehension.

## Conclusion

In women with refractory overactive bladder, use of a novel patient decision aid was associated with uniformly low decisional conflict and positive patient feedback. PDAs appear to be valuable tools for supporting shared decision-making in those with refractory OAB. Further studies are required to assess whether the reduction in initial decisional conflict translates to improved long-term treatment adherence and reduced posttreatment regret.

## Data Availability

The authors are happy to provide data upon reasonable request.

## Appendix

Link to PDA:

https://www.supportincontinence.org/wp-content/uploads/2025/03/Interactive-Overactive-Bladder-PDA_v0.2_11MAR2025.pdf
